# Supplementary material for: Certainty in Uncertain Times: Dental Education during the COVID-19 Pandemic–A Qualitative Study
Source: Int J Environ Res Public Health. 2023 Feb 10;20(4):3090. doi: 10.3390/ijerph20043090 (PMC9962035; doi:10.3390/ijerph20043090)
Supplement: Supplementary file 1 [file ijerph-20-03090-s001.zip › ijerph-2187395-supplementary.pdf]

Interview guide for lectures and students

1) How did you perceive teaching and learning as part of the switch to digital courses?

2) How did you perceive the implementation of the practical courses?

3) In your previous statements, in which moments did you feel safe?

4) In which moments did you feel more insecure?

5) What would be the background, a possible explanation that you describe it that way?

6) What conditions would you like to have that would make you feel more comfortable/safe in such a situation?

7) Is there anything you could spontaneously name right now that would help you feel more confident?
